# Cardioprotective Effect of Hydroalcohol Extract of Andaliman (*Zanthoxylum acanthopodium* DC.) Fruits on Doxorubicin-Induced Rats

**DOI:** 10.3390/ph17030359

**Published:** 2024-03-10

**Authors:** Aminah Dalimunthe, Denny Satria, Panal Sitorus, Urip Harahap, Intan Farah Diba Angela, Syukur Berkat Waruwu

**Affiliations:** 1 Department of Pharmacology, Faculty of Pharmacy, Universitas Sumatera Utara, Medan 20155, Indonesia; uripharahap@usu.ac.id (U.H.); intan.fda@gmail.com (I.F.D.A.); 2 Department of Pharmaceutical Biology, Faculty of Pharmacy, Universitas Sumatera Utara, Medan 20155, Indonesia; dennysatria@usu.ac.id (D.S.); sitoruspanal@gmail.com (P.S.); 3 Faculty of Pharmacy and Health Sciences, Universitas Sari Mutiara Indonesia, Medan 20123, Indonesia; syukurbkwaruwu@gmail.com

**Keywords:** cardioprotective, doxorubicin, quercetin, *Zanthoxylum acanthopodium* DC.

## Abstract

Andaliman (*Zanthoxylum acanthopodium* DC.) fruit is a spice plant widely used in North Sumatra. The chemical content in the Andaliman plant has a cardioprotective effect, with antioxidant properties that inhibit oxidative stress and free radicals. SOD (superoxide dismutase), BNP (Brain Natriuretic Peptide), and cTnT (troponin T) are measured as markers of heart damage, and histopathology is to see heart damage. Quercetin administration was used as a comparison. The hydroalcoholic extract’s phytochemical content and chemical elements were analyzed using LC-HRMS and GC-MS. The findings showed that the hydroalcohol extract of Andaliman fruits affected the blood levels of SOD, BNP, and cTnT in the blood of doxorubicin-induced rats. SOD levels increased, and BNP decreased; the 300 mg/kg BW group was not significantly different from the 50 mg/kg BW quercetin group. cTnT levels also decreased; the 150 mg/kg BW and 300 mg/kg BW groups were not significantly different, and both were better than the 50 mg/kg BW quercetin group. EAF with 150 mg/kg BW and 300 mg/kg BW can also repair damage to rat heart tissue caused by doxorubicin. Andaliman fruit extract has cardioprotective effects and anti-free radical activity due to its content and potential to be developed.

## 1. Introduction

Cardiovascular disease is the most significant cause of death in the world and encompasses several cardiovascular diseases, including heart disease [1,2]. In 2019, it was estimated that 17.9 million people died from cardiovascular disease, which is 32% of all global deaths, and 85% of these deaths were caused by heart attacks and strokes [3]. Factors that cause heart disease include increased serum lipid levels, hypertension, smoking, and a diet high in saturated fat and cholesterol [4,5,6]. Apart from these factors, some drugs have the potential to cause cardiotoxicity, for example, cancer drugs, one of which is doxorubicin. Doxorubicin is a drug that was introduced in cancer therapy in the late 1960s. It has emerged as one of the most potent broad-spectrum antitumor anthracyclines. Doxorubicin can treat various types of cancer, including leukemia, lymphoma, soft tissue sarcomas, and solid tumors. Its cytotoxic effect is desirable on malignant cells; on the contrary, there is the potential for undesirable cardiotoxic effects [7,8,9,10,11], so cardiac protection is needed, which can contribute to heart preservation by reducing or even preventing heart damage [12,13,14]. The cardioprotective effects of several plants have recently been widely studied, one of which is to minimize the cardiotoxic potential of doxorubicin. Plant chemical compounds reported to have cardioprotective effects contain flavonoids and alkaloids [15,16,17,18].

Andaliman (*Zanthoxylum acanthopodium* DC.) fruit is a spice plant widely found in North Sumatra in traditional Batak cuisine [19,20]. Andaliman fruit contains flavonoids, terpene alkaloids, benzophenthridine alkaloids, pyrroloquinoline alkaloids, quaternary isoquinoline alkaloids, aporphyrine alkaloids, and the terpenoid group, namely geranyl acetate (35%). It is dominated by citrus aromas, namely limonene and citronellol. Other components are β-myrcene, β-ocimene, linalool, and E-1-decenal [21,22]. Andaliman ethyl acetate extract contains alkaloids, flavonoids, glycosides, saponins, tannins, and steroids [23]. Traditionally, Andaliman is used to cure stomachaches and toothaches and stimulate appetite. Reports on its pharmacological effects show that Andaliman ethyl acetate extract has cardioprotective effects and anti-free radical activity [24,25].

The content of Andaliman fruit, which contains flavonoids, is related to antioxidant properties. It has activity in inhibiting oxidative stress by inhibiting the formation of free radicals in male rats induced by doxorubicin [26,27]. Superoxide dismutase (SOD), as an endogenous antioxidant, influences levels of oxidative stress, while troponin T is a parameter of heart damage, followed by Brain Natriuretic Peptide (BNP) as a marker of damage to the ventricles [28,29]. Therefore, this research was conducted to test the hydroalcohol extract of Andaliman fruits (EAF) for its cardioprotective activity and its effect on SOD, BNP, and troponin T (cTnT) levels. Histopathology was performed to see damage to the heart organ. Quercetin was used as a comparison because of its antioxidant and cardioprotective properties, which are helpful against cardiovascular disease [30]. The phytochemical constituent analysis of the extract was also carried out using LC-HRMS, and chemical element analysis was performed using GC-MS.

## 2. Results

### 2.1. Phytochemicals Constituent

**Figure 1:**
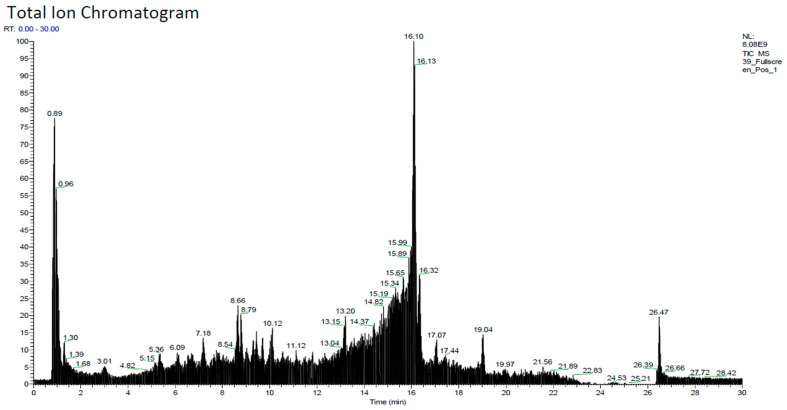
LC-HRMS chromatogram of hydroalcohol extract of *Zanthoxylum acanthopodium* DC.

Analysis of the phytochemical content of the hydroalcoholic extract was determined to obtain information on the compounds contained in EAF using LC-HRMS. The results are given in Table 1, and the chromatogram is in Figure 1.

### 2.2. Chemical Constituents of Hydroalcohol Extract

**Figure 2:**
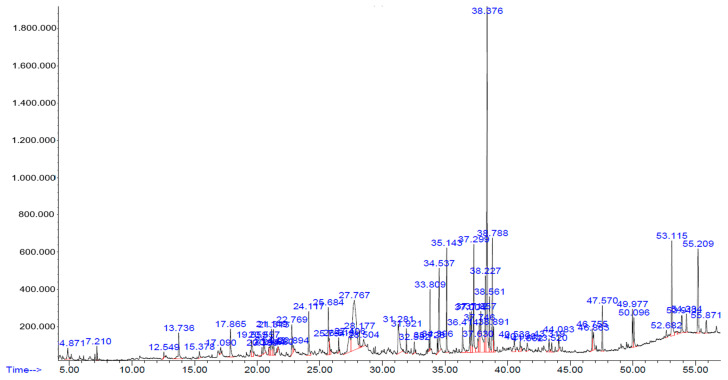
GC-MS chromatogram of hydroalcohol extract of *Zanthoxylum acanthopodium* DC.

Analysis of chemical compounds of hydroalcohol extract with gas chromatography and mass spectrometry resulted in 20 significant compounds. The analysis of chemical compounds of hydroalcohol extract with GC-MS is shown in Figure 2 and Table 2.

### 2.3. Results of SOD, BNP, and cTnT Measurements

**Figure 3:**
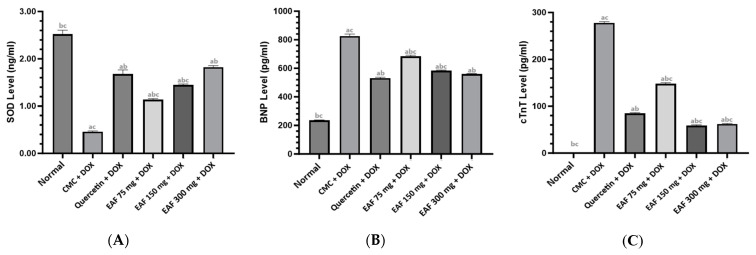
The levels of SOD (**A**), BNP (**B**), and cTnT (**C**). EAF: hydroalcoholic extract of Andaliman fruits; DOX: doxorubicin; (a) significantly different from the normal group (*p* < 0.05); (b) significantly different from the CMC + DOX group (*p* < 0.05); (c) significantly different from the quercetin 50 mg/kg BW + DOX group (*p* < 0.05).

The results of SOD, BNP, and cTnT measurements can be seen in Figure 3. The highest SOD levels occurred in the normal group with a mean value of 2.52 ng/mL, while the lowest SOD levels were in the CMC + DOX (CMC Sodium 0.5% + doxorubicin) group with a mean value of 0.46 ng/mL followed by the lowest to highest values; the EAF 75 mg/kg BW + DOX group, EAF 150 mg/kg BW + DOX group, quercetin 50 mg/kg BW group, and EAF 300 mg/kg BW + DOX group had mean values of 1.13 ng/mL, 1.44 ng/mL, 1.68 ng/mL, 1.82 ng/mL, respectively. The EAF 75 mg/kg BW + DOX group had SOD levels that were still higher than the CMC + DOX group, meaning that a dose of 75 mg/kg BW + DOX already had the effect of increasing SOD levels in doxorubicin-induced rats. Other results showed that the 150 mg/kg BW + DOX group had a higher SOD increasing effect than the 75 mg/kg BW + DOX EAF group, while the 300 mg/kg BW + DOX EAF group had a higher SOD increasing effect than the EAF 150 mg/kg BW + DOX group. The EAF 300 mg/kg BW + DOX group was the same as the 50 mg/kg BW + DOX quercetin group. The test results in this study for the EAF group, which had the best SOD-increasing effect, was EAF 300 mg/kg BW.

The highest BNP levels were In the CMC + DOX group with a mean value of 825 pg/mL, while the lowest BNP levels were in the normal group with a mean value of 237.96 pg/mL, followed by the lowest to highest values in the quercetin 50 mg/kg BW, EAF 300 mg/kg BW and EAF 150 mg/kg BW, and EAF 75 mg/kg BW + DOX groups, respectively, with mean values of 531.36 pg/mL, 559.32 pg/mL, 582.64 pg/mL, and 683.14 pg/mL. The normal group was significantly different from the other groups, meaning that the administration of doxorubicin increased BNP levels. The CMC + DOX group, which is the carrier group, looked significantly different from the other groups, meaning that administration of doxorubicin without quercetin and EAF increases BNP levels; the administration of quercetin and the test substance EAF provided activity to reduce BNP levels in rats induced by doxorubicin, and the effect of giving CMC as a carrier still showed an increase in BNP due to doxorubicin administration. The quercetin 100 mg/kg BW + DOX group was significantly different from the standard groups, EAF 75 mg/kg BW + DOX and EAF 150 mg/kg BW + DOX (*p* < 0.05), but not significantly different from the EAF 300 mg/kg BW + DOX group. The decrease in BNP levels in rats in the EAF 300 mg/kg BW group was the same as the quercetin 50 mg/kg BW + DOX group (*p* > 0.05).

Research shows that the group of rats given doxorubicin experienced an increase in cTnT levels. The highest cTnT levels were in the CMC + DOX group with a mean value of 278.11 pg/mL, while the lowest cTnT levels were in the EAF 150 mg/kg BW and EAF 300 mg/kg BW groups with a mean value of 58.95 pg/mL, respectively, and 62.09 pg/mL. The next highest order was the quercetin 50 mg/kg BW and EAF 75 mg/kg BW groups. The EAF 150 and 300 mg/kg BW groups had better cTnT reduction activity than the 50 mg/kg BW quercetin group. There was a significant difference that occurred between the standard group and the other groups, meaning that the administration of doxorubicin increased cTnT levels. The CMC group, as a carrier, apparently still had increased cTnT levels. This evidence states that CMC as a carrier does not affect EAF activity in reducing cTnT levels. The quercetin and 75 mg/kg BW + DOX group significantly differed from the other groups. The 150 mg/kg BW + DOX group was not significantly different from the 300 mg/kg BB + DOX group, meaning they had a significant effect on the CMC + DOX group, and that EAF 75 mg/kg BW + DOX had cTnT reducing activity. The EAF 75 mg/kg BW group, which had the smallest dose, showed reduced cTnT activity compared to the CMC + DOX group. The three doses of EAF in this study were tested, and the results showed that the doses of EAF 150 mg/kg BW + DOX and EAF 300 mg/kg BW + DOX provided the best cTnT-reducing activity.

### 2.4. Cardiac Histopathology Results

**Figure 4:**
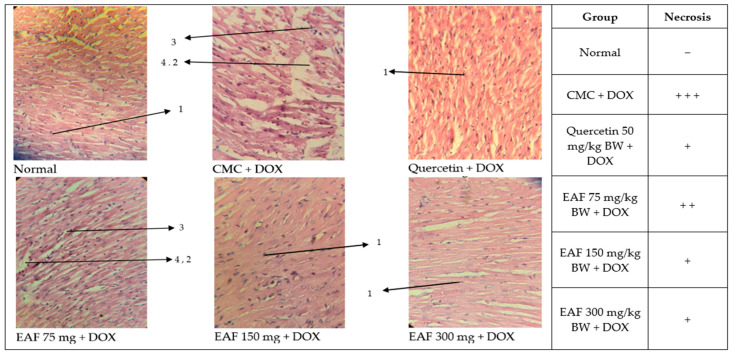
Histopathological evidence of the protective effect of hydroalcohol extract of Andaliman fruits from cardiotoxicity caused by doxorubicin (40× magnification). (1) Normal myocardial cells; (2) necrosis; (3) pyknosis; (4) karyolysis; (−) normal myocardial cells; (+++) severe necrosis; (++) moderate necrosis; and (+) mild necrosis.

The results of measuring immunological parameters such as SOD, BNP, and cTnT have been discussed. The rat heart organs were examined with Hematoxylin and Eosin (HE) staining, and necrosis cells were visible, meaning there was damage to myocardial cells, providing confirmation from the results of immunological parameters. The results of cardiac histology can be seen in Figure 4.

The average group showed normal cardiac morphology, while CMC + DOX showed more cardiomyocytes without nuclei and pyknosis karyolysis or severe necrosis. The EAF group at 75 mg/kg BW showed a moderate increase in cardiomyocytes without nuclei. Pyknosis and karyolysis also appeared moderate. The EAF 75, 150, and 300 mg/kg BW groups showed a good improvement in cardiomyocyte damage caused by doxorubicin, as seen in the CMC + DOX group without an extract, meaning that EAF had a protective effect on cardiac cardiomyocytes against the damage caused by doxorubicin. The 150 and 300 mg/kg BW groups had a good effect on cardiomyocyte protection; here, it can be seen that the cells had low pyknosis and were orderly.

## 3. Discussion

Doxorubicin, via the oxidative stress pathway, increases the activity of the cardiac enzyme superoxide dismutase due to the reduction in free radicals from doxorubicin-semiquinone, which can cause heart damage [8,31,32,33,34]. This study tested EAF for BNP levels to determine the heart damage caused by doxorubicin and its effect on the ventricles. BNP is a marker of ventricular dysfunction. BNP levels increase if there is ventricular dysfunction [35,36,37,38]. The administration of doxorubicin can also cause cardiotoxicity, increasing troponin (cTnT) levels. Troponin is a regulatory protein and part of the contractile mechanism of the cardiac muscle. Troponin is bound within the filaments of the contractile apparatus. Troponin T binds to tropomyosin and actin. When cardiac myocytes are damaged, troponin is released into the circulation, causing troponin T levels to increase [15,39,40,41].

The secondary metabolites contained included alkaloids, flavonoids, glycosides, saponins, tannins, and steroids, which are thought to have exogenous antioxidant properties. Other compounds contained in the hydroalcohol extract of Andaliman fruits include β-myrcene, limonene, β-ocimene, linalool, citronellal, β-citronellol, neral, geraniol, geranial, geranyl acetate, and sesquiterpene (Table 1). It has been reported that the main compound found in Andaliman is geranil [42]. Geranil has an antioxidant effect, so it is also thought to be an exogenous antioxidant that helps increase SOD in rats induced by doxorubicin [43,44,45,46]. The decrease in BNP by administering EAF is thought to be caused by the compounds geranyl acetate and citronellal contained therein [47]. The flavonoids contained in Andaliman were reported by researchers, but if we compare the flavonoids contained in the same class of Andaliman, namely *Zanthoxylum Zanthoxyloides*, this plant flavonoid has been reported to contain glycosylated flavanones (eriocitrin) in fruit extracts [48].

Andaliman itself has been shown to have anti-free radical activity [25]. Endogenous free radicals, such as reactive oxygen or nitrogen species, occur in physiological functions in cell signaling and defense against microbes but can also harm cells, especially in ischemia/reperfusion injury. Free radicals, with their unpaired electrons, produce hydroxyl [49,50,51]. DNA causes the prevention of micromolecule synthesis, the formation of reactive oxygen species (ROS), DNA binding and cross-linking, and DNA damage [52,53,54]. The group given doxorubicin can damage cardiomyocytes, as seen in Figure 4 in the CMC + DOX, quercetin 50 mg/kg BW, EAF 75, 150, and 300 mg/kg BW groups, which is different from the normal group that consists of normal myocardial cells. Doxorubicin produces free radicals, which are bound by antioxidants, such as superoxide dismutase. These antioxidants are said to be protective because they bind free radicals [49]. Andaliman also has antioxidant properties from the substances contained in it. The EAF 75 mg/kg BW group has seen improvements in cardiomyocyte fragmentation, but cardiomyocytes without nuclei are still visible. The EAF 150 and 300 mg/kg BW groups appeared to be in better condition when compared to the CMC + DOX and EAF 75 mg/kg BW groups.

**Figure 5:**
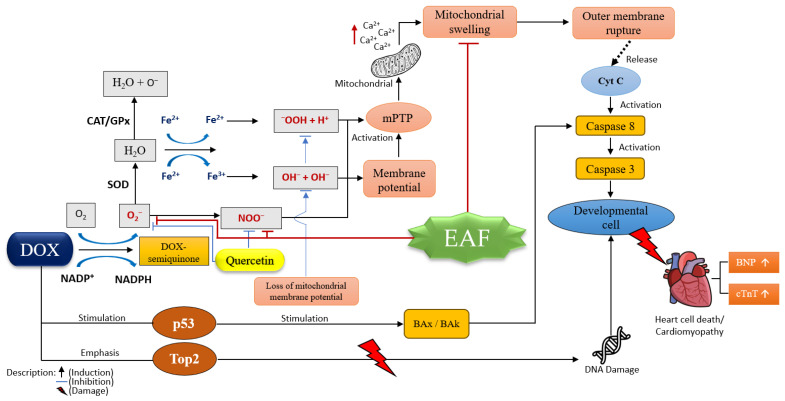
Proposed EAF mechanism.

EAF was able to increase SOD levels in rats induced by doxorubicin. SOD levels in the EAF 300 mg/kg BW group were not significantly different from those in the 50 mg/kg BW quercetin group. EAF was also able to reduce BNP levels induced by doxorubicin in rats. BNP levels for EAF rats in the 300 mg/kg BW group were not significantly different from the quercetin 50 mg/kg BW group, which was the group that was the best at reducing BNP. The results of cTnT measurements showed that EAF could reduce cTnT levels in rats induced by doxorubicin. The EAF 150 mg/kg BW and 300 mg/kg BW groups did not differ significantly in reducing cTnT, and both were better than the quercetin 50 mg/kg BW group. The histopathological observations of heart tissue showed that the EAF 150 mg/kg BW and 300 mg/kg BW groups could repair damage with equally mild degrees of necrosis. The EAF results improved the condition of heart tissue damage caused by doxorubicin. The proposed EAF mechanism is shown in Figure 5.

## 4. Materials and Methods

### 4.1. Plants, Tools and Chemicals

The Andaliman fruit used in this research was taken from Onan Runggu, Samosir Regency, North Sumatra. Andaliman fruit was identified at the Medanense Herbarium (MEDA) of the Universitas Sumatera Utara with the number 918/MEDA/2022. The tools used in this research were surgical instruments, oral sondes, laboratory glassware, animal scales, analytical balance (Mettler Toledo, Greifensee, Switzerland), a 1 mL syringe (OneMed, Surabaya, Indonesia), a 3 mL syringe (OneMed, Indonesia), blender (Phillips, South Jakarta City, Indonesia), drying cabinet, mortar and stamper, microtube, light microscope (Zeiss, Oberkochen, Germany), deck glass (Onemed, Indonesia), object glass (Onemed, Indoneisa), and ELISA reader (Thermo Scientific, Waltham, MA, USA). Meanwhile, the materials used in this research were doxorubicin, 0.5% NaCl, 10% formalin, quercetin, ELISA Kit SOD (ELK Biotechnology, Denver, CO, USA), ELISA Kit BNP (Elabscience, Houston, TX, USA), and ELISA Kit cTnT (Elabscience, USA).

### 4.2. Preparation of Extract of Andaliman Fruit

Fresh Andaliman fruit was washed thoroughly to remove soil and other impurities, then drained and weighed to obtain the wet weight. Next, the fruit was dried in a drying cupboard until dry. The dried simplicia was weighed and then blended into powder. Then, was placed in a plastic bag and stored at room temperature. The extract was made by maceration, namely Andaliman simplicia powder in a specific ratio, soaked in 60% ethanol. A total of 1.5 kg of simplicia powder with a suitable degree of fineness was placed into a vessel, poured with 11.25 liters of 60% ethanol, covered, and left for five days protected from light while being stirred repeatedly. After five days, it was filtered, and the dregs were squeezed. The dregs were washed with 60% ethanol, stirred, and mixed until 15 liters were obtained. The macerate was collected in a closed vessel, left in a cool place protected from light for two days, and then poured off. The extract was concentrated using a rotary evaporator and then dried using a freeze-dryer [55,56].

### 4.3. Phytochemicals Constituent Analysis with LC-HRMS

Phytochemicals from the extract were analyzed with TSQ exactive (Thermo) (LSIH, Brawijaya University). A total of 10 mg of the extract was dissolved in methanol with grade LC-MS/MS and vortexed (2000 rpm) for 2 min; the solution was then filtered with a 0.22 µm filter membrane using mobile phase A (0.1% formic acid in water and B (0.1% formic acid in acetonitrile) with gradient method and flow rate of 40 µL/minute. The elution system that was used was a gradient system with the following composition of the mobile phase: 0–2 min (5% B), 2–15 min (60% B), 15–30 min (5% B) for the column using Hypersil GOLD aQ 50 × 1 mm × 1.9 µm and the time for analysis was 30 min. The MS ionization source was ESI (+) with a Q-orbitrap mass analyzer. The *m*/*z* range was from 150 to 1000, with resolving power at 70,000 FWHM. The results were analyzed, and their compounds were discovered with *m*/*z* cloud software [57,58].

### 4.4. Analysis of Chemical Constituents by GC-MS

The extract was analyzed with GC-MS (Thermo) gas chromatograph with a fused silica capillary column (TG-5MS, 30 m × 0.25 mm, film 0.25 µm) using helium as a carrier gas with a flow rate of 1.02 mL/minute and with temperature programming from 70 °C for 5 min to 280 °C and an increase in temperature at 5 °C/min. The injector temperature was set at 280 °C. The mass spectrometer was performed using an interface temperature of 280 °C and an electron impact ionization of 70 eV with a scan mass range of 40–500 *m*/*z* (sampling rate 1.0 scan/s [59].

### 4.5. Animals and Study Design

Male Wistar rats weighing 180–200 g were housed in standard conditions and had free access to food and water. The animal use procedure obtained permission and guidance from the Animal Research Ethics Committee, Faculty of Mathematics and Natural Sciences, Universitas Sumatera Utara, with letter number 0564/KEPH-FMIPA/2022. Animals were divided into six groups (each consisting of four experimental animals).

Group I: Normal control test animals were not given any treatment, but food and drink were still provided.
Group II: Negative control test animals were given 0.5% sodium-CMC suspension.
Group III: Positive control test animals were given 50 mg/kg BW quercetin.
Group IV: test animals were given EAF at 75 mg/kg BW.
Group V: test animals were given EAF at 150 mg/kg BW.
Group VI: test animals were given EAF at 300 mg/kg BW.

**Figure 6:**
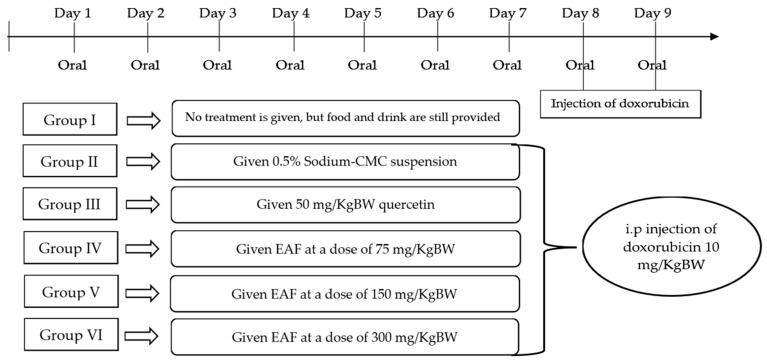
Timeline schedule of the treatment regimen.

All treatments (except normal controls) were administered once a day for seven consecutive days. On days 8 and 9, doxorubicin was administered at a dose of 10 mg/kg BW intraperitoneally, 1 h after administering the preparation. The timeline schedule of the treatment regimen can be seen in Figure 6.

The doxorubicin dose used in this study was based on our previous study [60]. A short-term rat model receiving high-dose doxorubicin injections would be suitable for assessing acute cardiotoxicity [61]. However, this study could not verify myocardial damage, so we performed histology on the heart.

### 4.6. Preparation of Blood Serum and Cardiac Organs

Blood sampling was conducted after the rats had fasted for 12 h or on the 10th day. Rats were anesthetized using ketamine, then dissected, and blood was taken using a 1 mL syringe directly from the rat’s heart up to ±5 mL; this was placed into a microtube and left for ±30 min. The blood was centrifuged at 3000 rpm for 20 min to obtain rat blood serum. The heart organ was also taken and placed in a pot containing formalin.

### 4.7. Measurement of Superoxide Dismutase, Brain Natriuretic Peptide, and Troponin T

SOD, BNP, and cTnT measurements were carried out using the ELISA method, which is a biochemical test that uses antibodies and enzyme-mediated color changes to detect the presence of antigens (proteins, peptides, hormones). A microplate reader carried out measurements using a plate kit obtained from ELK Biotechnology and Elabscience [62].

#### 4.7.1. SOD Measurement Procedure

A total of 0.1 mL of standard solution with a concentration of 10, 5, 2.5, 1.25, 0.63, 0.32, and 0.16 ng/mL was added to the standard well. A total of 0.1 mL of diluent solution was added to the control well. Then, 0.1 mL of the sample was added to the sample well. Then, the plate was closed and incubated at 37 °C for 80 min. The cover was opened, and the plate’s contents were removed; then, the plate was washed three times with 200 µL wash solution for washing each well. A total of 0.1 mL of the biotinylated antibody working solution was inserted into the standard sample and control wells at the bottom without touching the walls. The plate was closed and incubated at 37 °C for 50 min. The cover was opened, and the plate was washed thrice with a wash solution and left in the well for 1 min each time. A total of 0.1 mL of Streptavidin-HRP working solution was added to each well; then, the plate was closed and incubated at 37 °C for 50 min. The cover was opened, and the plate was washed five times with a wash solution; each time it was washed, the solution was left in the well for 1 min. A total of 90 µL of the TMB substrate was added to each well; then, the plate was closed and incubated at 37 °C in the dark for 20–30 min. A blue color was visible; after that, 50 µL of the stop reagent was added to each well and mixed thoroughly until the color changed to yellow. Absorbance was measured at 450 nm with a microplate reader immediately after adding the stop solution.

#### 4.7.2. BNP Measurement Procedure

A total of 50 µL of standard solutions with various concentrations of 2000, 1000, 500, 250, 125, 62.5, and 31.25 pg/mL was added to the standard well. A total of 50 µL of the buffer solution was added to the control well. Then, 50 µL of the sample was inserted into the sample well. A total of 50 µL of biotin-labeled antibody working solution was inserted into the standard, sample, and control wells at the bottom without touching the walls. Then, the plate was closed and incubated at 37 °C for 45 min. The cover was opened, and the plate’s contents were removed; then, the plate was washed three times with wash buffer with 350 µL of wash solution per well for 1 min per wash. A total of 100 µL of HRP conjugated working solution was added to each well; then, the plate was closed and incubated at 37 °C for 30 min. The cover was opened, and the plate was washed five times with a wash buffer; each time it was washed, the buffer was left in the well for 1 min. A total of 90 µL of the reagent substrate was added to each well; then, the plate was closed and incubated at 37 °C in the dark for 15–30 min. The blue color was visible. After that, 50 µL of the stop solution was added to each well and mixed thoroughly until the color changed to yellow. Absorbance was measured at 450 nm with a microplate reader immediately after adding the stop solution.

#### 4.7.3. cTnT Measurement Procedure

A total of 50 µL of standard solutions with various concentrations of 1000, 500, 250, 125, 62.5, 31.25, and 15.63 pg/mL was added to the standard well. 100 µL of buffer solution was added to the control well. Then, 100 µL of the sample was inserted into the sample well. The plate was closed and incubated at 37 °C for 90 min. The liquid was then removed from the well and was not washed. A total of 100 µL of biotin-labeled antibody working solution was inserted into the standard, sample, and control wells at the bottom without touching the walls. Then, the plate was closed and incubated at 37 °C for 60 min. The cover was opened, and the contents of the plate were removed; then, the plate was washed three times with a wash buffer with 350 µL of wash solution per well for 1 min per wash. A total of 100 µL of HRP conjugated working solution was added to each well; then, the plate was closed and incubated at 37 °C for 30 min. The cover was opened, and the plate was washed five times with wash buffer; each time the buffer was washed, the buffer was left in the well for 1 min. A total of 90 µL of the reagent substrate was added to each well; then, the plate was closed and incubated at 37 °C in the dark for 15–30 min. The blue color was visible. After that, 50 µL of stop solution was added to each well and mixed thoroughly until the color changed to yellow. Absorbance was measured at 450 nm with a microplate reader immediately after adding the stop solution.

### 4.8. Histopathological Observation

Cardiac histopathology was performed by cutting the organ using a sliding microtome with a thickness of 4 μm. The tissue incision was placed on a glass object and then on a hot plate at 37 °C. After drying, the glass objects were incubated at 37 °C for one night. The incision was rehydrated and stained with Hematoxylin and Eosin (HE) dye.

### 4.9. Data Analysis

Data were analyzed using an ANOVA test to determine differences between treatments. If there were differences, we continued using Turkey’s Post Hoc test to determine which variables differed. Based on the significance value, *p* < 0.05 was considered significant.

## 5. Conclusions

Based on the observations made, it can be concluded that the hydroalcohol extract of Andaliman fruits can influence the levels of SOD, BNP, and cTnT in the blood of rats induced by doxorubicin. The hydroalcoholic extract from Andaliman fruits can also repair damage to rat heart tissue caused by doxorubicin. However, further research must determine the compounds involved in this cardioprotective activity.

## Figures and Tables

**Table 1:** Phytochemical constituent analysis of hydroalcoholic EAF with LC-HRMS.

N	Name	Formula	Molecular Weight	Retention Time (min)
1	3-[(2-phenyl-1H-imidazol-4-yl)methylene]-1,3-dihydro-2H-indol-2-one	C_18_H_13_N_3_O	287.10427	14.391
2	4,7,8-trimethoxyfuro[2,3-b]quinoline	C_14_H_13_NO_4_	259.0833	10.768
3	N,N-Dimethyltryptamine	C_12_H_16_N_2_	188.13068	5.157
4	Luotonin A	C_18_H_11_N_3_O	285.08876	15.777
5	Citral	C_10_H_16_O	152.11952	5.187
6	4-Coumaric acid	C_9_H_8_O_3_	164.04676	6.542
7	Quercetin	C_15_H_10_O_7_	302.04158	9.615
8	Nootkatone	C_15_H_22_O	218.16611	17.545
9	7,7-dimethyl-3-spiro(4,4,-dimethyl-2,6-dioxocyclohexyl)-1,2,3,4,5,6,7,8-octahydro-5-quinolinone	C_18_H_25_NO_3_	303.1797	8.645
10	5,7-Dihydroxy-2-(4-hydroxyphenyl)-6,8-bis[3,4,5-trihydroxy-6-(hydroxymethyl)tetrahydro-2H-pyran-2-yl]-4H-chromen-4-one	C_27_H_30_O_15_	594.15666	5.707
11	Isorhamnetin	C_16_H_12_O_7_	316.05708	11.129
12	Berberine	C_20_H_17_NO_4_	335.11437	9.951
13	Ferulic acid	C_10_H_10_O_4_	194.05724	18.404
14	(-)-Caryophyllene oxide	C_15_H_24_O	220.18181	11.753
15	8,8-dimethyl-2H,8H-pyrano[3,2-g]chromen-2-one	C_14_H_12_O_3_	228.07764	13.956
16	Jasmonic acid	C_12_H_18_O_3_	210.12242	5.209
17	Kaempferol	C_15_H_10_O_6_	286.04656	10.929
18	Luotonin F	C_18_H_11_N_3_O_2_	301.0837	13.011

**Table 2:** Percentage of Zanthoxylum acanthopodium DC compounds.

N	Compounds	Retention Time (min)	Area (%)
1	Geraniol	17.862	1.24
2	2-methoxy-4-vinylphenol	19.564	1.10
3	Geranic acid	20.559	0.76
4	Geranyl acetate	21.315	0.87
5	6-hydroxy-3,7-dimethyl-2,7-octadienyl acetate	25.689	1.47
6	2,2,4-trimethyl-1,3-pentanediol di isobutyrate	26.520	0.35
7	Alpha cadinol	28.171	0.59
8	N,N-dimethyltryptamine	31.284	2.92
9	2,6,10-dodecatrien-1-ol,3,7,11-trimethyl acetate	31.927	0.70
10	Hexadecanoid acid	33.805	1.48
11	n-hexadecanoid acid	34.536	3.50
12	9,12,15-octadecatrienoic acid	37.107	1.60
13	Phytol	37.296	3.57
14	9,12-octadecadienoic acid	37.750	1.82
15	Lineloic acid ethyl ester	38.229	2.91
16	Furo[2,3-b]quinoline,4,7,8-trimethoxy	41.569	0.45
17	(+)-Sesamin	53.113	4.25
18	Stigmasterol	54.285	0.63
19	Gamma sitosterol	55.205	3.52
20	Beta amyrin	55.873	0.59

## Data Availability

Data are contained within the article.
